# The Piano-Forte

**Published:** 1864-07

**Authors:** 


### THE PIANO-FORTE.

THE very name brings with it associations of the sweetest, dearest, tenderest kind; it makes us think of sisters in the far past, whose voices have long since been hushed in death, and have gone upward to strike the strings of immortality; of daughters in their youth and beauty and loveliness; of home with all its affectionate and refining and elevating influences. In fact, we can scarcely think of a home without a piano. How it binds a family together! keeping the girls from the contaminations of the theater and the ball-room, and the boys from the street and the negro minstrelsy; cultivating a taste for all that is pure and gentle and loving, and, to that extent, throwing a barrier around the young; guarding them against the indulgence of vicious propensities and intemperate habits; implanting in their minds a lofty looking-down on low associations, and the companionship of the reckless, the dissolute, and the unrestrained. It is well then to know of an instrument which has such high social influences, that its value, more than that of any other musical instrument, depends on the quality of its tone, which, in turn, owes its perfection to one mechanical point, in reference to which that very valuable paper, recognized the world over as authority in the mechanic arts, *The Scientific American* says, under the head of the "Use and Abuse of the Piano-Forte":

"The great desideratum aimed at, by the best manufacturers of pianos, is to make them stand in tune well, for unless they succeed in this respect, the quality of tone or beauty of finish they may impart to their pianos is comparatively of little value. To attain this desirable object, therefore, is the principal aim of our best makers; but few, however, succeed, and we will briefly state the reason. The steel pins that hold the wires of a piano are driven into a solid block of wood, and in order that this wood may retain a firm hold of the pin, and yet admit of its being turned by the hammer of the tuner, not only is great care and skill necessary in regard to the fitting of these pins, but it is absolutely requisite that the wood forming the 'pin-block' should be of the very best seasoned material. Now this 'seasoned' wood is best when prepared by out-door seasoning instead of by artificial means; but unfortunately this former method requires considerable capital to admit of so much dead stock, as it were, lying by. This large capital but few manufacturers have, and the result is, they have to use heat-dried wood, and the majority place wood thus seasoned in their pianos that will not stand the action of the hot-air furnaces in such general use in private houses. The consequence in such cases can be readily foreseen, the result being that a year or two's use so shrinks up the wood of the pin-blocks of those pianos in which this half-seasoned stuff is used, that the pins move in the block from every hard blow on the wires, and hence the piano will not stand in tune.

"So much for the injurious effects of this artificial heat on a piano, as far as its standing in tune is concerned. In reference to its effect on the 'action' of a piano, namely, the keys and machinery for striking the wires, the result is, that the heat warps the keys, loosens the hold of the great number of screws used in an action, in the wood, and thereby causes the keys to stick or rattle, as the case may be. Now, how to obviate these evils is the question, and the answer is, in the first place, only to purchase those pianos that are made of thoroughly seasoned wood, and of the best quality of materials generally, for such only are the cheapest pianos, no matter what their first cost may be; and, secondly, to keep your piano as much from the influence of the hot air of your house furnace as possible, for it injures the best made pianos, and almost renders those of inferior quality useless."

Some years ago we drew attention to this fact of the proper seasoning of the timber, as the reason why the Worcester piano gave out the same sweet musical tones at the end of fifteen years' constant family usage as on the day it came from the manufacturer's ware-rooms. Every piece of wood connected with the keys and strings was, at that early day, in Mr. Worcester's establishment, subjected to a seasoning process for from two to six years; hence they had a reputation in the damp climate of the South and the West-Indies not equaled by those of any other manufacturer in the land. Many piano-makers have failed because they had not the means, the time, and the high moral principle to procure the best materials; hence no one family ever made a second purchase; no father, having bought one for his own use, made a present of another to his newly married daughter. Such men ought to fail. Worcester never failed. His extensive establishment was never shut up for an hour by a strike, for he early and always made it a practice to employ the very best hands, and he wisely concluded that they deserved the very best wages, and the very best he always gives.

The Worcester piano, so superior before, is absolutely peerless now by a patented improvement—the maker's own discovery—which so lengthens the tones of the instrument as to excite the astonishment and admiration of the very best of living performers, who have enthusiastically testified as to this point. Gottschalk does not hesitate to say that a hundred per cent is added to the volume of the tone by means of Worcester's hinged plate improvement; and Pattison, and Timm, and Maretzek, and Stein, and Mason, and Sanderson, and a long list of the most celebrated performers and most accomplished teachers join in the testimonial. As this important improvement is peculiar to Worcester's pianos, parents who are anxious to have the very best article for their children will obtain these unrivaled instruments in preference to all others, especially as they are afforded on as liberal terms as at other first-class establishments, with the additional most important advantage that the care and time and money spent in most manufactories in New-York, Philadelphia, and Boston in making a flash appearance, irrespective of any inherent utility, are given at Worcester's to the substantial and lasting finish of the instrument.

